# Using Web-Based Continuing Education to Improve New Diagnoses of Alzheimer Disease in Claims Data: Retrospective Case-Control Study

**DOI:** 10.2196/72000

**Published:** 2025-05-22

**Authors:** Katie Lucero, Thomas Finnegan, Soo Borson

**Affiliations:** 1 Medscape, 283-299 Market Street, 2 Gateway Center - 4th Floor, Newark, NJ, 07102, United States, 1 2123016700; 2 Keck School of Medicine, University of Southern California, Los Angeles, CA, United States

**Keywords:** Alzheimer disease diagnosis, continuing medical education, real-world outcomes, physician confidence, web-based CME, CME, self-efficacy

## Abstract

**Background:**

Alzheimer disease (AD) presents significant challenges to health care systems worldwide. Early and accurate diagnosis of AD is crucial for effective management and care to enable timely treatment interventions that can preserve cognitive function and improve patient quality of life. However, there are often significant delays in diagnosis. Continuing medical education (CME) has enhanced physician knowledge and confidence in various medical fields, including AD. Notably, web-based CME has been shown to positively influence physician confidence, which can lead to changes in practice and increased adoption of evidence-based treatment selection.

**Objective:**

This study investigated the impact of a targeted, web-based CME intervention on health care providers’ confidence, competence, and real-world outcomes in diagnosing early AD.

**Methods:**

The study employed a 2-phase design. Phase I used a pre-post assessment to evaluate immediate changes in knowledge and confidence before and after CME participation. Phase II involved a retrospective, matched case-control study to examine the impact of CME on AD diagnoses in claims data.

**Results:**

A 1-way ANOVA showed a significant effect of CME regarding change in the volume of AD diagnoses (*F*_1900_=5.50; *P*=.02). Compared to controls, CME learners were 1.6 times more likely to diagnose AD, resulting in an estimated net increase of 7939 new diagnoses annually. Post-CME confidence was associated with a greater likelihood of diagnosing AD (odds ratio 1.64; 95% CI 0.92-2.92; *P*=.09; n=219).

**Conclusions:**

Web-based CME participation is associated with increased real-world AD diagnoses. Findings offer a mechanism to explain the changes in clinical practice seen as a result of the CME intervention, which improves skills and confidence.

## Introduction

Alzheimer disease (AD) is a progressive neurodegenerative disorder that poses significant challenges to health care systems worldwide. AD affects more than 6.0 million persons in the United States, 7.9 million in Europe, and at least 50 million people worldwide [1,2]. The risk of AD increases with age. By 2050, the number of affected persons 65 years and older is expected to reach 12.7 million in the United States and over 152 million worldwide [1]. As the global population ages, the prevalence of AD has risen dramatically and will continue to rise, bringing new urgency to addressing the widespread lags in diagnosis that impede effective patient management and care.

Early diagnosis is vital for maintaining quality of life, delaying institutionalization, and improving treatment outcomes. Monoclonal antibodies that target plaque work best in the early stages of AD when pathologic changes are still relatively mild [3,4]. Early diagnosis allows for early initiation of treatment, which can help preserve patients’ functional abilities and cognitive function, thereby improving quality of life [5]. Early diagnosis can also reduce caregiver burden by helping patients and caregivers access culturally competent care and support services to improve quality of life [6].

Significant delays are common in the diagnosis and management of patients with AD. Physician practice patterns across several countries, including the United States, reveal that while approximately half of patients globally receive an AD diagnosis within 6 months of initial presentation, a significant number of patients remain undiagnosed for several months after initially presenting to a physician [7-9]. Misdiagnosis of AD in primary care settings is exceptionally high, with as many as two-thirds of patients being misdiagnosed [6]. While primary care physicians (PCPs) are typically first to see patients with mild cognitive impairment (MCI) and early AD [10], physician suspicion accounts for only 20% of AD diagnoses globally, with caregivers often serving as the primary impetus for seeking medical attention [8]. Overall, referral rates for specialist care are also low (14%‐23%) [8].

Recent updates to diagnostic and staging criteria for AD are based on biological indicators versus clinical syndrome [11]. In practice, AD is often diagnosed through the evaluation of cognitive symptoms, which is highly dependent on a clinician’s experience and skill [6]. In the absence of a single diagnostic test for AD, physicians rely on physical and neurological examination, mental status tests, imaging, and biomarkers for diagnostic purposes [12]. However, by the time a patient starts showing signs of cognitive impairment, underlying pathologic changes have likely been happening for a decade or longer [13].

Gaps in physician knowledge and insufficient specialized training partly drive challenges in the diagnostic process [12]. Physicians often struggle to distinguish normal aging from dementia, and between various types of dementia [14], and demonstrate limited awareness of early cognitive impairment indicators [8]. Notably, many physicians lack self-efficacy in diagnostic abilities, including their skills to detect signs of MCI and differentiate MCI from AD [12]. Physicians also lack self-efficacy to use and interpret cognitive testing and neuroimaging [5]. While specialists are more likely to use magnetic resonance imaging, PCPs often rely on computed tomography scans, which are less informative [12]. This lack of self-efficacy can lead to delayed diagnoses, hindering timely interventions and optimal patient outcomes.

Continuing medical education (CME), including web-based CME, has shown promise in improving physician knowledge and self-efficacy across various medical domains, including in AD diagnosis [15-17]. However, the relationship between improving knowledge, competence, self-efficacy, and real-world outcomes (RWOs) in AD diagnosis remains understudied [10,16-18]. Improving knowledge does not guarantee its application in practice. Rather, improving self-efficacy is an essential intermediary between knowledge and practice change. Self-efficacy, a motivational construct also known as confidence, empowers physicians to act upon their knowledge and implement learned skills (also known as competence) [19]. However, the relationship is not strictly linear. Improvements in and reinforcement of knowledge and competence can also increase self-efficacy, which in turn influences practice change [20,21]. These relationships suggest that clinicians with a greater sense of self-efficacy following CME activities demonstrate a stronger intention to change their practice, regardless of whether they improved their knowledge [22,23].

This study investigates whether a targeted, web-based CME intervention can improve physicians’ self-efficacy, competence, and RWOs in diagnosing early AD. The study addressed the following hypotheses: (1) competency scores for HCPs will increase; (2) the proportion of HCPs who are confident will increase; and (3) the volume of new AD diagnoses will increase for the CME group compared with the matched control group.

## Methods

### Study Design

#### Overview

**Figure 1.**
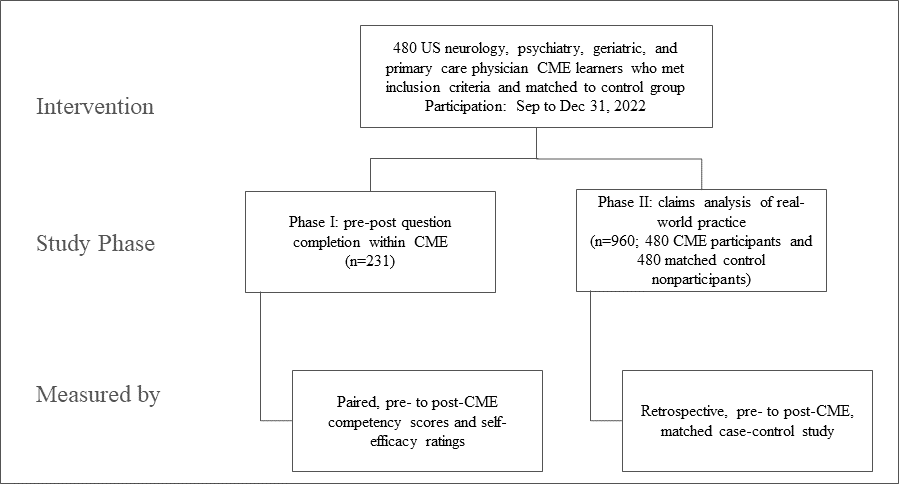
Study design. CME: continuing medical education.

We conducted 2 study phases using the outcomes assessment framework by Moore et al [24] to assess leading indicators (changes in competency scores and confidence ratings) and lagging indicators of success (changes in real-world performance, specifically the volume of new AD diagnoses). Phase I focused on AD diagnosis within CME activities. Phase II focused on AD diagnosis in the real world (Figure 1).

#### Phase I: Educational Assessment

We employed a paired, pre-post design to assess the impact of CME activities on knowledge and competency scores and confidence ratings immediately before and after the point of learning in the activity for learners from September 13 to December 31, 2022.

#### Phase II: Real-World Outcomes

We conducted a retrospective, matched case-control study from March 2022 to June 2023 to evaluate the impact of CME activities on diagnosing patients with AD. The intervention period spanned from September 13 to December 31, 2022, with data collection extending from March 2022 to June 2023, assessing practice 6 months before and 6 months after the CME participation date (“index date”).

#### CME Intervention

The intervention consisted of a web-based CME initiative for PCPs and neurologists designed to improve competence and confidence in early recognition and diagnosis of AD. The first activity focused on best practices in delivering care for patients with AD in primary care and neurology (released September 13, 2022, through September 13, 2023) [25]. The activity was valid for a maximum of 0.50 American Medical Association Physician Recognition Award (AMA PRA) Category 1 Credit. Topics included triaging and assessing patients with cognitive impairment in primary care, coordinating with neurologists, and treatment goals. A subsequent series of 3 simulated online office visits focused on increasing clinicians’ ability to identify and communicate with patients experiencing early cognitive impairment (released October 14, 2022, through October 14, 2023, valid for a maximum of 0.25 AMA PRA Category 1 Credit) [26]. Each office visit centered around an interactive patient-physician vignette for which a decision on screening and evaluation was required. Vignettes included White and Black patients with cognitive impairment due to MCI, cognitive impairment due to early AD, and cognitive impairment not due to dementia. Participants previewed the chief concern for each patient vignette via a landing page with an interactive, graphic table of contents. The activity required participants to investigate cognitive complaints and select diagnostic tests. Faculty feedback was included in each activity.

### Inclusion Criteria

Physicians were included if they participated (that is, viewed the content, after the front matter and disclosures) in at least 1 activity in the study period, practiced in the United States, had at least 1 patient who met the inclusion criteria, and had complete claims data available for the study period. Patients were included if they were at least 60 years of age and saw the learner during the study period as evidenced by at least 1 *International Statistical Classification of Diseases and Related Health Problems, Tenth Revision* (*ICD-10*) code or prescription by the learner.

### Sample

Medscape member registration provides the country of residence, profession, specialty, and National Provider Identifier (NPI; if applicable) number for health care providers (HCPs). A total of 1725 US physicians in the target specialties with a valid NPI were learners between September 13 and December 31, 2022. Of these, 1310 had matches with claims data with at least 1 patient who met the inclusion criteria. The RWO intervention group (RWO learners) comprised 480 physicians who met the full inclusion criteria, participated in the CME activities during the intervention period, and were matched to a nonparticipant control (287/480, 60% PCPs; 100/480, 21% neurologists; 71/480, 15% psychiatrists; and 22/480, 5% geriatric specialists). Of the 480, 231 fully completed an activity.

### Matching Process

An equal number of 480 HCPs who did not participate in CME served as the control group. We used a 1:1 matching ratio to pair cases with controls. The matching criteria included: (1) number of patients with AD, (2) number of patients diagnosed with AD by the HCP, (3) profession, (4) specialty, and (5) the first 2 digits of the HCP’s ZIP code. Match-It in R was used to match the propensity score by the number of patients with AD and the number of patients diagnosed with AD by the HCP. Exact matching was used for profession, specialty, and the first 2 digits of the ZIP code. An independent samples *t* test showed no statistically significant difference in the number of patients with AD seen in the preperiod by the CME and matched control groups (*t*=−0.24; *P*=.81).

### Real-World Data Collection

We sourced real-world data from Medscape licensed claims data, accessed in November 2023. This dataset provided comprehensive information on patient visits, diagnoses, and procedures performed by the participating HCPs. Data were aggregated at the patient level, and patient counts were aggregated at the HCP level. See Multimedia Appendix 1 for the codes accepted as indicators of diagnosis.

### Measures

Three primary outcome measures assessed the effectiveness of the CME intervention: (1) competency score, (2) confidence rating, and (3) number of patients newly diagnosed with AD by the HCP. Additionally, we examined whether there was an association between being confident following CME and real-world diagnoses.

#### Competency Score

Competency scores were assessed by asking 3 case-based vignette questions pre- and postpoint of learning in the CME activity. Scores were aggregated at either the learning topic or activity level and then to the HCP. Scores ranged from 0% to 100%. Questions were developed to assess learning against learning objectives related to cognitive assessment or differentiation between MCI and AD. Questions were developed by content experts and reviewed by an expert AD HCP, an outcomes assessment specialist, and a copyeditor.

#### Self-Efficacy Rating

A question assessed self-efficacy on a 5-point Likert-type scale, with higher scores indicating greater self-efficacy (eg, “How confident are you right now in your ability to assess patients for cognitive impairment?”). Learners were deemed “confident” if they rated themselves as a 4 or 5. We used the term “confident” because it is more easily understood by the respondent than self-efficacy and use “confident” in the methodology and results for ease of interpretation when referring to those who select a 4 or 5.

#### New AD Diagnoses

New AD diagnoses were assessed by examining patient-level *ICD-10* data. Patients with an AD *ICD-10* code by the HCP of interest in the timeframe of interest were identified. Then, the patient’s history 2.5 years prior was examined to assess whether they had previously received any AD *ICD-10* codes by any HCP. If there was no history of receiving an AD *ICD-10* code previously, the patient’s *ICD-10* code was considered a new AD diagnosis by the HCP of interest. The count of patients who met these criteria was aggregated at the HCP level.

### Statistical Analysis

#### Phase I: Educational Assessment

We assessed immediate changes in learner competency scores and confidence via 4 matched pair questions before and after CME participation. The McNemar test evaluated change in the proportion of learners who rate themselves as confident. The McNemar test was chosen because it measures differences in paired proportions against the null hypothesis. A paired samples *t* test was conducted to measure mean differences in paired samples. Overall competency changes were assessed using paired samples *t* tests. Statistical significance was set at *P*<.05 for all tests.

#### Phase II: Real-World Outcomes

The relationship between CME participation and the postintervention volume of new AD diagnoses was assessed with a 1-way ANOVA. One-way ANOVA was chosen because we wanted to examine the change in volume of AD diagnosis from pre– to post–index date (dependent variable) and whether being a learner was associated with this change (independent variable with 2 independent groups). The dependent variable was the change in the volume of AD diagnoses from pre– to post–index date, and the independent variable was CME participation (versus control). In a secondary analysis, we explored the association between postintervention confidence (confident=1, not confident=0) and AD diagnosis (diagnoser=1, nondiagnoser=0) via logistic regression.

The association between being confident and diagnosing AD was explored because the more we know about mechanisms for change that we can immediately measure at scale within a web-based CME activity, the more effective our education can be. A dichotomous independent variable and dichotomous dependent variable were selected because the research question focused on whether confidence predicts being a diagnoser. More confidence should not equate to diagnosing more because how many patients get diagnosed depends on the types of patients an HCP sees. Previous research used this same dichotomy and found similar results [25].

Statistical significance was set at *P*<.05 for all tests, and analyses were performed using SAS version 9.4 (SAS Institute).

### Ethical Considerations

The Sterling Institutional Review Board deemed this study exempt under the terms of the US Department of Health and Human Service’s Policy for Protection of Human Research Subjects at 45 CFR §46.104(d) [27]. The ethical standards of the Declaration of Helsinki were applied to all research procedures. As the study was exempt, there was no requirement for informed consent. The institutional review board approval covered secondary analysis without additional consent. The data were deidentified prior to analysis to safeguard participant information. No compensation was provided to participants.

## Results

### Phase I: Competency and Confidence

“Completers” answered all linked questions within at least 1 of the CME activities, representing 48% (231/480) of the larger learner population. After participation, RWO learners demonstrated a 34 percentage point increase in correct answers for competency in the diagnosis of AD (33% prescore to 67% postscore; *P*=.008) and a 16 percentage point pre- to postactivity increase in the proportion of those who were confident in assessing cognitive function and diagnosing AD (75/231 preactivity to 99/231 postactivity; *P*<.001).

### Phase II: New AD Diagnoses

ANOVA showed a significant effect of CME regarding change in the volume of AD diagnoses (*F*_1900_=5.50; *P*=.02). The 6-month postactivity increase in new AD diagnoses was 160% greater for the CME group than the control group, as verified by claims data. RWO learners diagnosed 239 more patients after education (487 diagnoses pre-education vs 726 diagnoses posteducation). Control-group learners diagnosed 91 more patients after education (517 diagnoses pre-education vs 608 diagnoses posteducation). Neurologists had the highest increase in new AD diagnoses (1.58 per neurologist), while psychiatrists had the lowest (0.10 per psychiatrist). The logistic regression model showed a trend within the CME group toward a significant positive relationship between being confident in AD assessment post-CME and diagnosing AD in the real world in the 6 months following CME (odds ratio [OR] 1.64, 95% CI 0.92-2.92; *P*=.09; n=219). Table 1 summarizes the RWO learners and the matched control group on key outcomes from claims data.

**Table 1.** Number of patients with Alzheimer disease (AD) and number of patients newly diagnosed with AD before and 6 months after the activity.

	Patients with AD, mean (SD)		Patients newly diagnosed with AD, mean (SD)	
	Pre	Post	Pre	Post
CME^a^ (n=480)	2.16 (7.51)	2.33 (8.35)	1.01 (3.60)	1.51 (5.59)
Geriatric specialists (n=22)	2.27 (5.23)	2.64 (5.95)	1.41 (2.92)	2.05 (4.36)
Neurologists (n=100)	6.50 (15.03)	7.14 (16.79)	3.04 (7.17)	4.62 (11.29)
PCPs^b^ (n=287)	1.12 (2.38)	1.14 (2.43)	0.51 (1.14)	0.71 (1.52)
Psychiatrists (n=71)	0.20 (0.60)	0.27 (0.81)	0.10 (0.38)	0.20 (0.62)
Control (n=480)	2.05 (6.27)	1.92 (6.03)	1.08 (3.33)	1.27 (4.07)
Geriatric specialists (n=22)	1.95 (4.36)	1.86 (2.92)	0.82 (1.62)	1.00 (1.69)
Neurologists (n=100)	5.94 (12.11)	5.50 (11.40)	3.24 (6.40)	3.71 (7.46)
PCPs (n=287)	1.15 (2.56)	1.08 (3.03)	0.57 (1.42)	0.68 (2.31)
Psychiatrists (n=71)	0.24 (0.60)	0.31 (1.04)	0.17 (0.48)	0.27 (1.03)

a CME: continuing medical education.

b PCP: primary care physician.

## Discussion

### Principal Findings

This matched case-control study examined the impact of a web-based, vignette-based CME on participants’ knowledge, competence, self-efficacy, and RWOs in diagnosing early AD. Participation in CME was associated with a significant (*P*=.02) increase in the diagnosis of early AD. RWO learners were more likely to be diagnosers than control-group physicians, with a magnitude of increase in AD diagnoses that was 1.6 times higher for RWO learners than control-group physicians. The estimated net increase of 7939 in new AD diagnoses in the year following participation for CME learners through the expiration of the activities for credit indicates a substantial positive impact of education on AD diagnosis rates. RWO learners also improved their confidence in identifying early forms of AD (*P*<.001). When HCPs were confident after CME, they had a 1.64 greater odds of diagnosing AD.

### Comparison with Prior Work

Research suggests that CME can effectively improve physician knowledge, self-efficacy, and competence regarding dementia care in general. A large study in Australia evaluated an accredited CME program on the diagnosis and management of dementia in primary care. Participants who completed the program reported feeling significantly more confident in their knowledge, skills, and ability to provide care for people with dementia [15,16]. Our study not only affirms the impact of CME on real-world AD diagnoses but also offers a mechanism to explain the changes in real-world practice seen as a result of the CME intervention. Previous research shows that improvements in knowledge and competence following CME participation are associated with increased self-efficacy, and posteducation self-efficacy mediates the relationship between knowledge and competence and intention to change [20,21]. A recent secondary analysis of knowledge, competency, self-efficacy, and clinical practice using pre- and postparticipation data from web-based CME interventions in 3 different therapeutic areas combined with medical claims data examined the relationship between knowledge, competency, self-efficacy, and real-world clinical practice [23]. Knowledge and competency (*P*=.08; OR 1.515, 95% CI 0.953-2.410) and self-efficacy (*P*<.001; OR 2.768, 95% CI 1.705-4.492) were significant predictors of clinical practice. However, the effect size for self-efficacy was larger, suggesting that clinicians confident in their abilities were more likely to utilize evidence-based treatments. These results suggest that self-efficacy plays a significant mediating role in influencing clinical practice.

Reinforcement of existing knowledge also appears to influence clinical practice. A study that examined the relationships among knowledge, competence, self-efficacy, and intention to change across 57 online oncology-certified education programs published from 2018 to 2020 found that both improvements in and reinforcement of knowledge and competence are significant predictors of changes in self-efficacy [20]. Lucero et al [28] supported this finding. They found that participants who reinforced their knowledge had higher posteducation confidence ratings than participants who improved their knowledge after controlling for posteducation scores. Reinforcement of knowledge also likely explains why neurologists demonstrated the most significant increase in the number of new AD diagnoses.

### Limitations

Potential confounding factors could affect the relationship between CME participation and increased AD diagnoses. Physicians who participated in CME may have been more motivated to improve their practice, potentially leading to increased diagnoses regardless of the CME content. Three activities were case-based simulated patient visits and 1 was a video-based discussion on cases. We did not tease out which activities might have been more or less impactful and whether participation in multiple activities was associated with practice change. Concurrent initiatives, such as other AD awareness campaigns or participation in non–study-related CME activities focused on AD or cognitive disorders during the study period, could also have confounded results. The control group also saw an increase of 91 more diagnoses postintervention, suggesting some external factors may have influenced diagnosis rates. Professional learning occurs in many places, given the demands of clinical practice and the requirement to maintain licensure. While changes for the control group were anticipated, matching based on demographic and practice factors helps reduce biases associated with those factors such as opportunity to diagnose, training, types of patients seen, and environment in which one practices.

Despite these limitations, the comprehensive matching strategy minimizes potential confounding factors and ensures group comparability. By matching profession, specialty, and ZIP code, the study controls for some differences in baseline knowledge, experience, and practice patterns associated with different medical specialties, as well as variations in patient demographics and health care access. Matching based on the number of patients with AD controlled for differences in patient population and exposure to AD cases. Using a time-aligned control group helped to control temporal factors, such as concurrent initiatives, that would affect both groups equally. Results were assessed by counting claims nested in patients to better tease out patients with their first AD diagnosis from a learner versus a nonlearner physician. Using paired pre- and postintervention data for individual learners enhances the statistical precision of the analysis, reducing sampling error and providing a robust assessment of the education’s impact. Including the matched control group at follow-up increases confidence that changes are associated with education. Future research should explicitly examine how CME interventions affect AD diagnosis rates across different racial and ethnic groups and identify with more detail the mechanisms for change. We identified self-efficacy as a mechanism for practice change, but we should further understand which components in the CME influenced self-efficacy.

### Conclusion and Significance

Diagnostic delays contribute to suboptimal patient outcomes in AD. By using a matched case-control design and assessing both immediate educational outcomes and subsequent changes in diagnostic behavior, this study provides evidence for the potential of CME as a tool to increase AD diagnosis. This web-based CME intervention increased participant likelihood of diagnosing AD, led to a greater number of new AD diagnoses than the control group, and fostered a positive relationship between postintervention confidence and diagnosis rates. Building self-efficacy should be a key objective in education interventions with practice-changing potential, alongside improving, reinforcing, and validating existing knowledge. Overall, this study shows the power of real-world data in demonstrating the impact of CME on clinical behavior and offers a first step in identifying CME’s impact on dementia care. We are currently conducting a second phase of this initiative. Future directions could include a breakdown of CME engagement levels and learning outcomes by specialty to clarify which provider groups benefit most from this intervention.

## Supplementary material

10.2196/72000 Multimedia Appendix 1 Codes used for the study.

## Abbreviations

AD: Alzheimer disease
AMA PRA: American Medical Association Physician Recognition Award
CME: continuing medical education
HCP: health care provider
*ICD-10*: *International Statistical Classification of Diseases and Related Health Problems, Tenth Revision*
MCI: mild cognitive impairment
NPI: National Provider Identification
OR: odds ratio
PCP: primary care physician
RWO: real-world outcome
