# Supplementary material for: Using Web-Based Continuing Education to Improve New Diagnoses of Alzheimer Disease in Claims Data: Retrospective Case-Control Study
Source: JMIR Med Educ. 2025 May 22;11:e72000. doi: 10.2196/72000 (PMC12121534; doi:10.2196/72000)
Supplement: Multimedia Appendix 1 [file mededu-v11-e72000-s001.docx]

# Multimedia Appendix: Codes used for the study

**“Physical” for inclusion**: PROC:99213, PROC:99214, PROC:99203, PROC:99204, PROC:99212

**Alzheimer’s diagnosis**: ICD:G30, ICD:G308, ICD:G300, ICD:G301, ICD:G310, ICD:G309
